# Prediction of hematocrit through imbalanced dataset of blood spectra

**DOI:** 10.1049/htl2.12006

**Published:** 2021-04-06

**Authors:** Cristoforo Decaro, Giovanni Battista Montanari, Marco Bianconi, Gaetano Bellanca

**Affiliations:** ^1^ Department of Engineering University of Ferrara Ferrara Italy; ^2^ MISTER Smart Innovation Bologna 40129 Italy; ^3^ CNR‐IMM‐UOS di Bologna and MISTER Smart Innovation Bologna Italy; ^4^ Department of Engineering University of Ferrara Ferrara Italy

## Abstract

In spite of machine learning has been successfully used in a wide range of healthcare applications, there are several parameters that could influence the performance of a machine learning system. One of the big issues for a machine learning algorithm is related to imbalanced dataset. An imbalanced dataset occurs when the distribution of data is not uniform. This makes harder the implementation of accurate models. In this paper, intelligent models are implemented to predict the hematocrit level of blood starting from visible spectral data. The aim of this work is to show the effects of two balancing techniques (SMOTE and SMOTE+ENN) on the imbalanced dataset of blood spectra. Four different machine learning systems are fitted with imbalanced and balanced datasets and their performances are compared showing an improvement, in terms of accuracy, due to the use of balancing.

## 1 INTRODUCTION

There is a great interest on optical investigation of blood: this technique could enable rapid and continuous monitoring of blood parameters during ex vivo treatments, such as dialysis [1, 2]. Some studies exploited empirical correlations to evaluate hematocrit [3], others made use of machine learning to measure relevant blood parameters such as oxygen saturation [4].

Machine learning has been successfully used in a wide range of healthcare applications. These include: the identification of skin cancer from images using intelligent model [5], or predict cardiovascular attack based on patients' characteristics, such as biometric data, clinical history and lab test results [6]. Many different applications of machine learning in medicine have demonstrated accurate results and machine learning and big data are able to detect diabetic retinopathy and diabetic macular edema in retinal funds photography with as accuracy as human physicians [7]. Machine learning are computational iterative models, which allow an algorithm to program itself, learning from a database of examples. These examples include observations, direct experiences, data from measurements and they are used in order to find the desired behaviour, without specifying mathematical or physical rules. Current ability to record massive amount of data has deeply changed healthcare and this has helped machine learning to find widespread applications in this field. Thanks to these big databases, machine learning algorithms are able to provide diagnosis [8] or to predict diseases [9].

Despite of its success in many areas from speech recognition [10] to autonomous driving vehicle [11], machine learning has encountered different impediments when applied to medicine. Medicine presents unique challenges and machine learning often is not able to provide results which improve clinical cares. These obstacles are mainly due to the impossibility to have large and high quality data in order to correctly train the algorithms. In other cases, data are sufficient, but they do not represent the entire possible scenario or are not uniformly distributed [8]. Databases with non uniform data dramatically reduce the effectiveness of machine learning; these collections of data are known as imbalanced datasets.

Different studies [12, 13, 14] have proposed strategies to face the problem of class imbalance in biomedical data. Each of these works exploit the use of different balancing techniques in order to increase the performance of the models.

The aim of this work is to show the higher performance of machine learning using two balancing techniques, synthetic minority oversampling technique (SMOTE) and SMOTE with edited nearest neighbour (SMOTE+ENN), on the prediction of hematocrit from an imbalanced dataset of spectral measurements of blood. Four different machine learning techniques (ridge regression, elastic net, random forest, artificial neural network) are trained with imbalanced and balanced datasets and the results of different predictions are then compared. These machine learning models were selected because they showed highest accuracy from previous investigations and studies [15].

The two balancing techniques, SMOTE and SMOTE+ENN, have been chosen, among other possible approaches, as an effective machine learning improvements on imbalanced dataset of blood spectra for the prediction of hematocrit. Other balancing techniques can be exploited as, for example, Adasyn. Adasyn is an improvement of SMOTE which introduces some variances to the synthetic data, in order to increase the variability. It can be considered as a valid alternative to confirm the validity of the proposed approach on blood data analysis when imbalanced datasets occur.

This paper is organized as follows: Section 2 shows the setup used for collecting spectral data of blood and gives a basic clinical background; Section 3 introduces the theoretical problem of imbalanced dataset and the classical approaches for balancing datasets. Section 4 describes the machine learning algorithms with all the hyper‐parameters chosen after a fine tuning process. In Section 5, the composition of data is analysed, while Section 6 shows the methodology proposed in this work. The results are shown and commented in Section 7. Finally, Section 8 presents the conclusions.

## 2 SETUP FOR DATA COLLECTION

**FIGURE 1:**
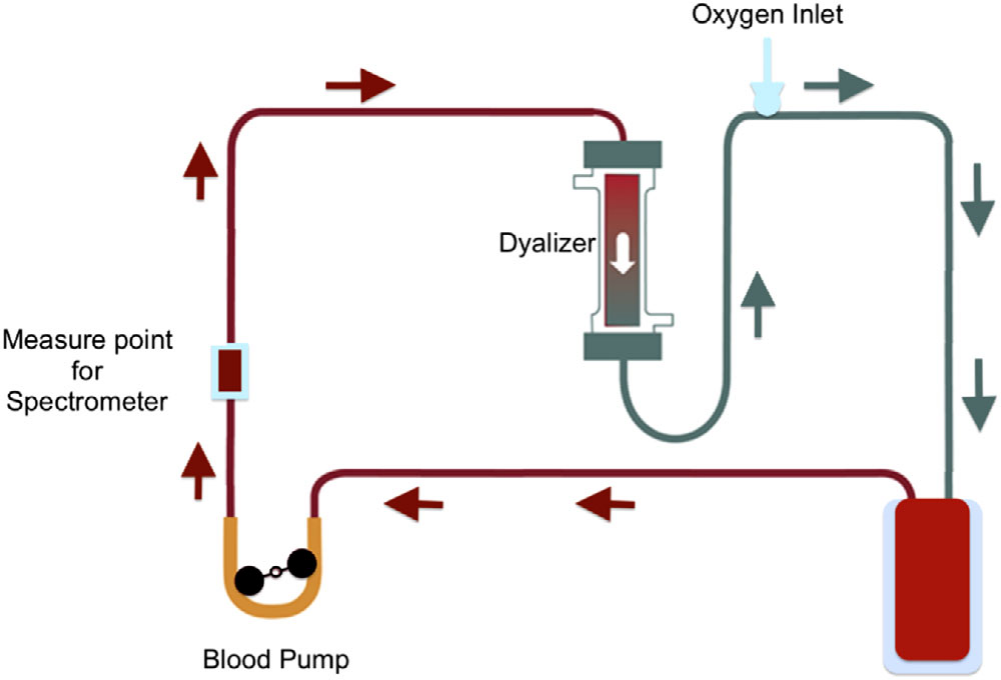
Operational setup for simulating ex vivo dialysis treatment

Machine learning techniques are applied for the evaluation of hematocrit based on animal blood visible spectra which are obtained with the setup and methodology described in [15]. Figure 1 illustrates the experimental setup for simulating dialysis treatment: the blood flows through the tube circuit and the spectra are recorded without stopping the flow. The application of machine learning for prediction of hematocrit could have a huge impact on human treatment of hemodialysis: the continuous monitoring of hematocrit can be time‐saving for patients and can avoid complications such as hypotension, muscle cramps and lightheadedness [16]. People who suffer of renal diseases are subjected to hemodialysis treatment to purify the blood. The treatment should be repeated four times a week per patient and it is a long treatment which is performed until the hematic parameters, such as hematocrit, reach standard values [17]. Hematocrit (or Hct) is the ratio between corpuscular part of blood volume and its total volume; its standard unit is percentage. During data collection, dialysis sessions have been performed on animal blood using a rigorous methodology. These sessions are simulations of a real treatment which is performed in vivo on human patients. The target level of hematocrit is usually measured through centrifugation: a capillary tube is filled with blood sample and, after few minutes of centrifugation, the different parts of blood are well separated. It is then possible to evaluate the ratio between the red blood cells and the total volume. This is a standard medical measurement method to evaluate hematocrit and the results from centrifugation are considered the target value for machine learning algorithms. The setup used in this work has a dialyser which allows to modify the hematocrit levels of blood; the aim is to provide as uniform as possible samples to the machine learning algorithm. The blood used in this work is from bovines; these animals have an hematocrit level lower than human standard ones [18] and it has been difficult to store enough samples of hematocrit in standard human values, due to the chemical difference between animal and human blood. The result is an imbalanced dataset where most of spectra show hematocrit levels lower than standard.

## 3 IMBALANCED DATASET

An imbalanced dataset occurs when the distribution of target is not uniform among the different classes. Most of data in real world are often imbalanced, for example fraud detection datasets or spam mail detection datasets are commonly imbalanced: their samples are not equally represented. This problem is recurrent in different healthcare applications where machine learning is applied. For example, many available datasets of skin cancer images [8] are imbalanced: they have a different number of photo of non‐disease examples over disease samples. The different distribution of negative and positive examples makes harder the implementation of accurate models which are not able to generalize predictions. Moreover, the error related to the minority class is often critical, because the model misunderstands people who are really affected by disease. In terms of machine learning, the aim is to implement an automated model with highest accuracy and highest sensitivity, due to the importance of possible related consequence on human health. The sensitivity is the hardest challenge for automated machine algorithms when train data are imbalanced, because data belonging to the most frequent class have a negative effect on the predictions. Simple predictive accuracy is clearly not appropriate in such situations, while higher sensitivity and highly rate of correct detection in the minority class is more desirable. Different proposals have been provided to reduce the effects of imbalanced dataset on machine learning models [19, 20, 21]. There are two main different approaches: under sampling and oversampling. Undersampling involves a random removal of samples belonging to most frequent class [22]. The result is a more balanced dataset, but the data size becomes smaller. Therefore, undersampling is the best approach for big dataset, where removing some data cannot lead to loss of information. The second technique is oversampling; it involves the duplication of some data belonging to minor classes. Oversampling is the best choice with limited size datasets, but it produces overfitting; for this reason, the algorithm will not be able to implement a general model. Basic oversampling techniques generate overfitting, but there are more advanced approaches for balancing a dataset avoiding overfitting, for example SMOTE and SMOTE+ENN techniques. synthetic minority oversampling technique [22] is an advanced oversampling method, which creates synthetic samples in the minority class of imbalanced datasets. It avoids overfitting because data are not already present in the dataset. SMOTE was developed by Chawla in 2002 [23] who proposed, for the first time, an oversample of minority class by creating synthetic examples. SMOTE algorithm takes data from the minority class and introduce synthetic examples along the segments joining any of the minority class nearest neighbours. The steps for generating synthetic samples are:

- The algorithm considers the difference between the feature vectors and their nearest neighbours;
- It multiplies the difference by a random number between 0 and 1 and add it to the feature vector;
- A random point along the line segment between two specific features is then selected.

SMOTE forces the decision region of minority class to become more general and, consequently, more robust. In literature, there are other advanced techniques to balance datasets. These are a combination of SMOTE followed by cleaning data techniques [24], such as edited nearest neighbour [25]. ENN deletes all the misclassified data from training set using KNN optimization technique [26]. ENN removes all the misclassified samples; it optimally eliminates outliers and possible overlap samples among the different classes. The combined approach of SMOTE+ENN is promising for imbalanced dataset, as it improves the final accuracy of the model.

## 4 MACHINE LEARNING IMPLEMENTATION

In order to find the best machine learning model for the prediction of hematocrit, four different machine learning algorithms have been implemented with different training datasets and, then, compared with same test samples. In this paper, the investigated machine learning techniques are:

1. Ridge regression
2. Elastic net
3. Random forest
4. Artificial neural network

Ridge regression and elastic net are two linear regression approaches with different regularization techniques [27]. Regularization allows the implementation of more general models.

Random forest [27] is an ensemble method where many decision trees are trained together with a subset sample of observations. Finally, artificial neural networks (ANN) are data driven algorithms: they change their structures and connections based on information that flows through them. They are optimized to find out complex and non linear relationship between inputs and outputs. These four different algorithms are all optimized through hyperparameter optimization techniques. In linear models, different values of penalty factor are manually tested, to finally obtain the most accurate result. Moreover, a grid search is used to fine tune hyperparameters in random forest. The hyperparameter optimization of ANN is trickier, because there are a lot of parameters to take into account. Talos library [28] is used to perform a grid search with ANN. During grid search, different models are implemented with all possible combination of parameters, this technique stores each model and it finally evaluates the performance to find the parameters set which provides the best accuracy.

Tables 1, 2, 3 and 4 report the main hyperparameters with their optimized values. The code is developed in Python and the models are implemented using Scikit–Learn and Keras libraries. More details about each parameter are reported into Scikit–Learn [29] and Keras manuals [30].

**TABLE 1:** Principal hyperparameters used for Ridge Regression algorithm

Ridge regression	
alpha	0.1
tol	0.00001
max iteration	None
solver	auto

**TABLE 2:** Principal hyperparameters used for elastic net regression algorithm

Elastic net	
alpha	1
r	0.8
max iteration	10000
tol	0.0001
selection	cyclic

**TABLE 3:** Principal hyperparameters used for random forest algorithm

Random forest	
criterion	mse
# estimators	100
max depth	15
min samples split	2
min samples leaf	1
max features	log2

**TABLE 4:** Principal hyperparameters used for artificial neural network

Artificial neural network	
# hidden layer	1
# neurons in first hidden layer	16
activation function	elu
kernel initializer	normal
optimizer	Adam
epochs	2000

**TABLE 5:** Composition of imbalanced dataset

Imbalanced dataset	
# Total training samples	249
# Data in class 0	150
# Data in class 1	99

**TABLE 6:** Composition of SMOTE dataset

SMOTE dataset	
# Total training samples	300
# Data in class 0	150
# Data in class 1	150

**TABLE 7:** Composition of SMOTE + ENN dataset

SMOTE+ENN dataset	
# Total training samples	290
# Data in class 0	145
# Data in class 1	145

## 5 DATASET COMPOSITION

The dataset is composed by 293 different spectra of animal blood at different hematocrit levels. Each sample is composed by 287 values of absorbance spectral measures at different wavelengths in visible range. The spectral data represent the input features for machine learning, while the target is represented by the value of hematocrit, which was evaluated through standard techniques. This amount of data is enough to show the promising approach of machine learning for the prediction of hematocrit as reported in other study [15]. Moreover, the range of Hct is full exhaustive, because it covers both patients with standard Hct values and patients with renal disorders. A simple preprocessing normalization is used, before splitting the data into train and test set. Normalization changes the values of the features to a common scale, without distorting the ranges of values, in order to enhance the accuracy of machine learning. The dataset is then divided into train and test set, the first one is used to fit the parameters of the model. An imbalanced train dataset reduces the performance of the model, SMOTE and SMOTE+ENN can reduce this effect. Labels are added to the train set to divide the data into two classes:

1. Spectra with hematocrit level lower or equal to 35 belong to Class 0;
2. Spectra who belong to hematocrit greater than 35 belong to Class 1.

Three different training sets are then prepared:

- The original imbalance training set,
- The balanced training set where SMOTE is applied,
- The balanced training set obtained through SMOTE and ENN as data cleaning technique.

## 6 METHODS

**FIGURE 2:**
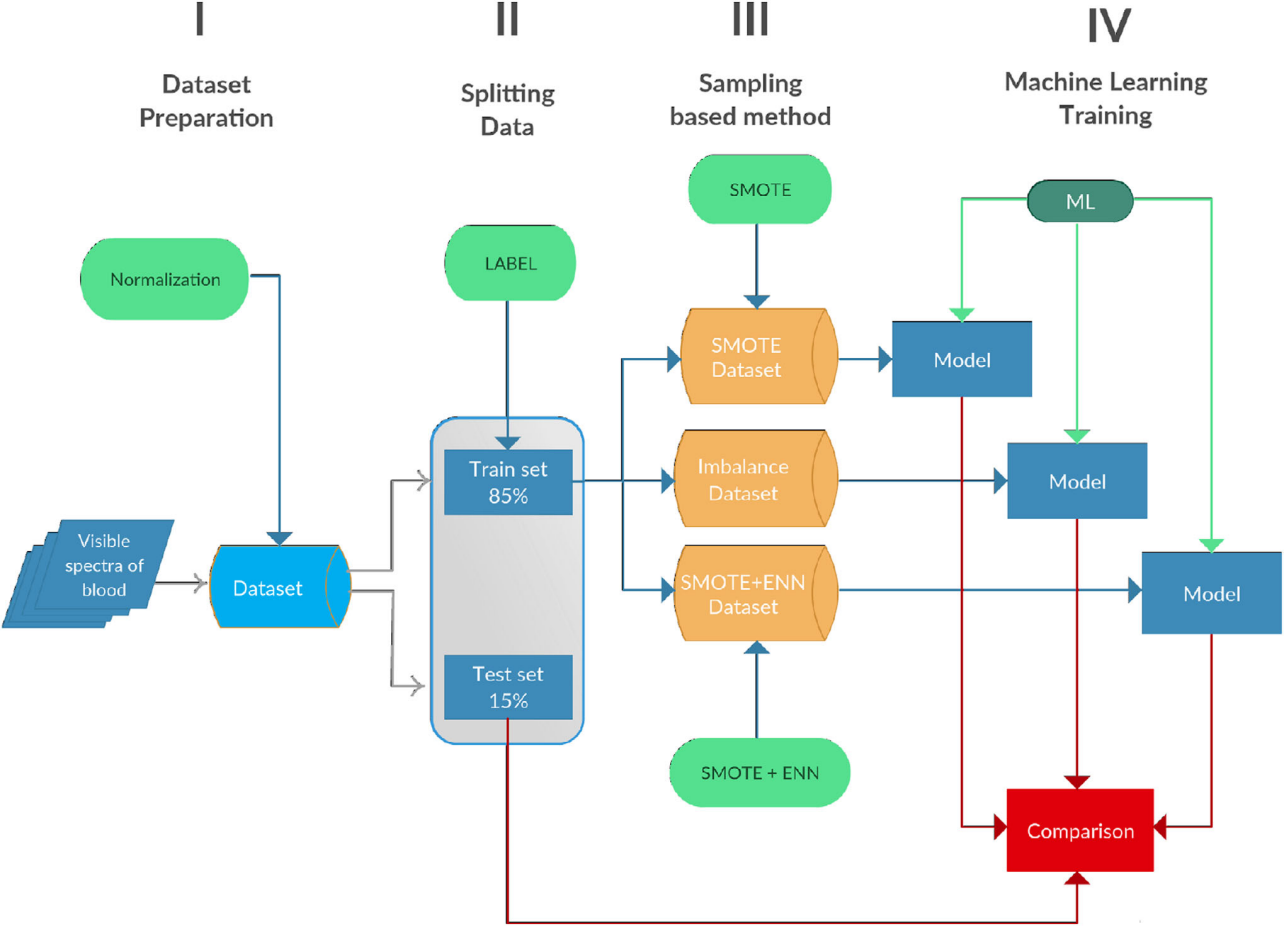
Block diagram describing the methodology considered in this paper

**FIGURE 3:**
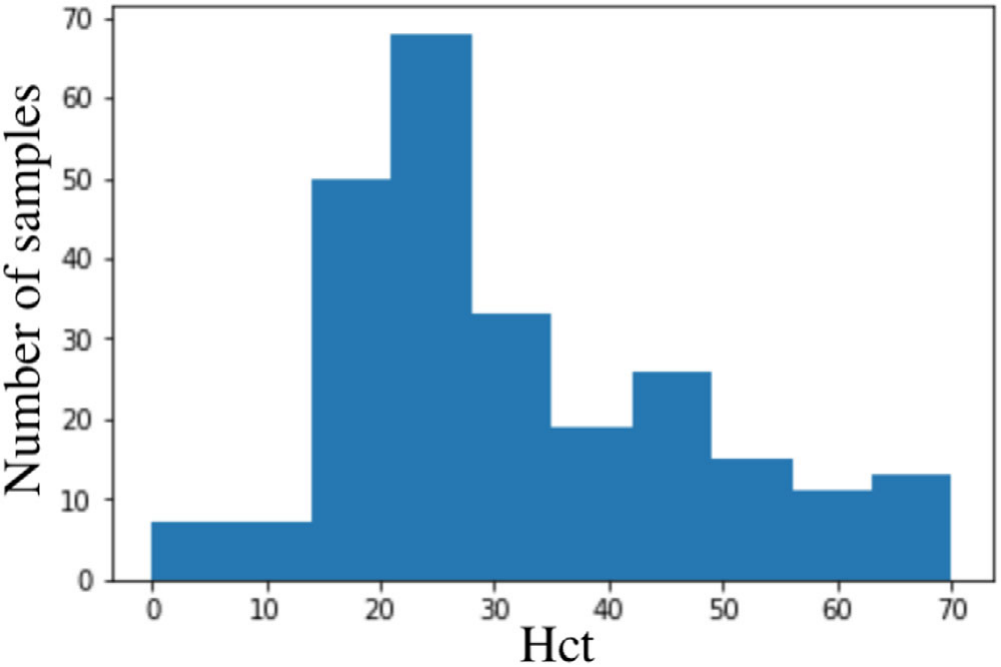
Distribution of samples in training set

The block diagram in Figure 2 summarizes the methodology proposed in this work. The data are collected and organized in a dataset. The dataset is scaled using Robust Scaler normalization [29] provided by Scikit–Learn library. After normalization, the dataset is randomly split in train and test datasets. The train dataset is composed by 249 samples; it represents the 85% of whole data, the remaining 15% representing the test set. The histogram in Figure 3 shows the distribution of samples in the training dataset, the samples are randomly extracted from the whole dataset. Some samples are under human minimum level of Hct, but they are not removed from the dataset, in order to increase the range of predictable Hct levels. The dataset is imbalanced: there are a lot of samples with hematocrit level close to 25%. Models are influenced by these data and it will be difficult to predict accurately samples with higher Hct. The training data are divide into two classes, that is assigned by choosing Hct threshold value equals to 35% as described in Section 5. The training set is consequently composed by: Class 0 represents the most frequent class, while class 1 has lower number of samples in it. The SMOTE and SMOTE+ENN are applied to the same training set. SMOTE and SMOTE+ENN methods are applied using Imbalanced‐learn library [31]. The SMOTE technique balances the data adding synthetic samples to class 1. The resulting dataset is composed by: while SMOTE+ENN dataset is composed by: The ENN algorithm has deleted five samples from each class (3.3% of total samples), because it operates a data cleaning which removes samples belonging to border class decision. The class labels are then removed and all datasets are trained. The comparison between the models involves evaluation of regression score function (*r* ^2^) and mean squared error (MSE) for all the models. Both parameters evaluate the error between desired and predicted values. However, MSE and *r* ^2^ give only a statistical evaluation of the overall error.

In classification tasks, there are different evaluation parameters, such as sensitivity or specificity. These evaluation metrics reflect more accurately the performance on imbalanced dataset than the standard ones, because they take into account of minority class. In regression, there are not advanced evaluation metrics focused on less frequent samples. Therefore, it can be convenient to measure the accuracy of the models using the standard evaluation parameters for regression (MSE and *r* ^2^ ) evaluating error on only less frequent data. A subset is generated; it includes all the data from test set with hematocrit over the threshold value (35), which is the standard hematocrit human level.

**FIGURE 4:**
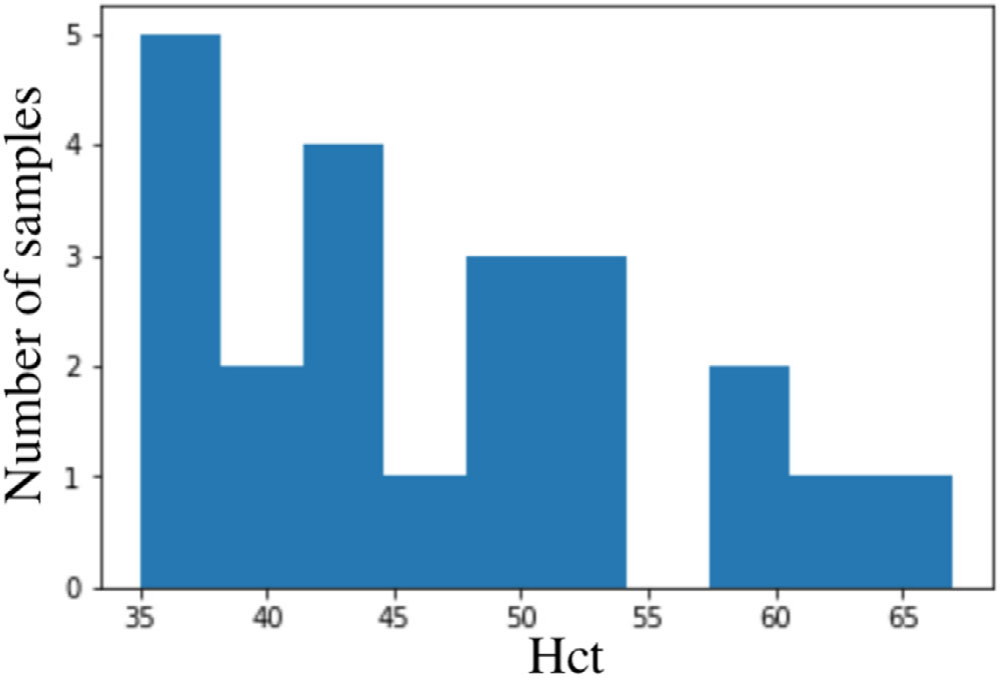
Test set with values within standard human range

The distribution of data belonging to this small test set is shown in figure 4. These samples will be predicted using all the fitted models and the results are statistically investigated.

It is crucial to focus on this subset of data, because during real dialysis treatment on ex vivo human blood most of data will be within this range.

## 7 RESULTS

The models are trained with all the three datasets and the performance of each model is evaluated on the same test set. Results are reported in Table 8:

**TABLE 8:** Performances of machine learning algorithms with evaluation metric results

Ridge regression		
	*r* ^2^	MSE
Imbalanced dataset	0.90	21.57
SMOTE	0.87	27.20
SMOTE + ENN	0.87	27.96

Elastic net		
	*r* ^2^	MSE
Imbalanced dataset	0.89	23.06
SMOTE	0.88	26.70
SMOTE + ENN	0.87	27.39

Random forest		
	*r* ^2^	MSE
Imbalanced dataset	0.82	37.30
SMOTE	0.76	52.13
SMOTE + ENN	0.77	49.56

Artificial neural network		
	*r* ^2^	MSE
Imbalanced dataset	0.92	16.81
SMOTE	0.93	15.01
SMOTE + ENN	0.95	11.11

Table 8 shows comparative performance results of different machine learning techniques fitted with different training dataset. Ridge regression and Elastic Net show close results; both linear models are very accurate, showing a small error and high *r* ^2^. Moreover, the balancing techniques do not increase the performance, because the model trained with imbalanced datasets shows lower MSE in both Ridge and Elastic Net. Despite of hyperparameter optimization, Random Forest shows lower performance than linear models. In this case, the best model in terms of *r* ^2^ and MSE is the one fitted with the imbalanced dataset. Moreover the performance on model trained with balanced datasets is very low. ANN is the most promising machine learning technique for prediction of hematocrit. The models are very precise with the highest *r* ^2^ and the lowest MSE among all the models. Figure 4 shows the linear regression plots of the models implemented by ANN techniques. They are trained with (a) SMOTE dataset and (b) SMOTE+ENN and represent the most accurate models, with a *r* ^2^ equals to (a) 0.93 and (b) 0.95. The test set has the same distribution of the training set: therefore, the models are fitted with training data similar to the test set. The result shows higher accuracy for models trained with imbalanced dataset. The results are different if the same statistical analysis is conducted on only tests that are within human standard range for hematocrit. Therefore, same statistical performance analysis is carried out on a smaller test set, where samples with hct in the range between 35 and 67 are considered. These results are reported in Table 9.

**TABLE 9:** Performances of machine learning algorithms focused on human standard range samples

Ridge regression on human range test set		
	*r* ^2^	MSE
Imbalanced dataset	0.72	22.46
SMOTE	0.65	28.08
SMOTE + ENN	0.64	29.26

Elastic net on human range test set		
	*r* ^2^	MSE
Imbalanced dataset	0.69	25.54
SMOTE	0.66	27.45
SMOTE + ENN	0.65	28.85

Random forest on human range test set		
	*r* ^2^	MSE
Imbalanced dataset	0.18	65.02
SMOTE	0.01	79.02
SMOTE + ENN	0.10	71.36

Artificial neural network on human range test set		
	*r* ^2^	MSE
Imbalanced dataset	0.72	23.25
SMOTE	0.86	12.87
SMOTE + ENN	0.85	13.49

**FIGURE 5:**
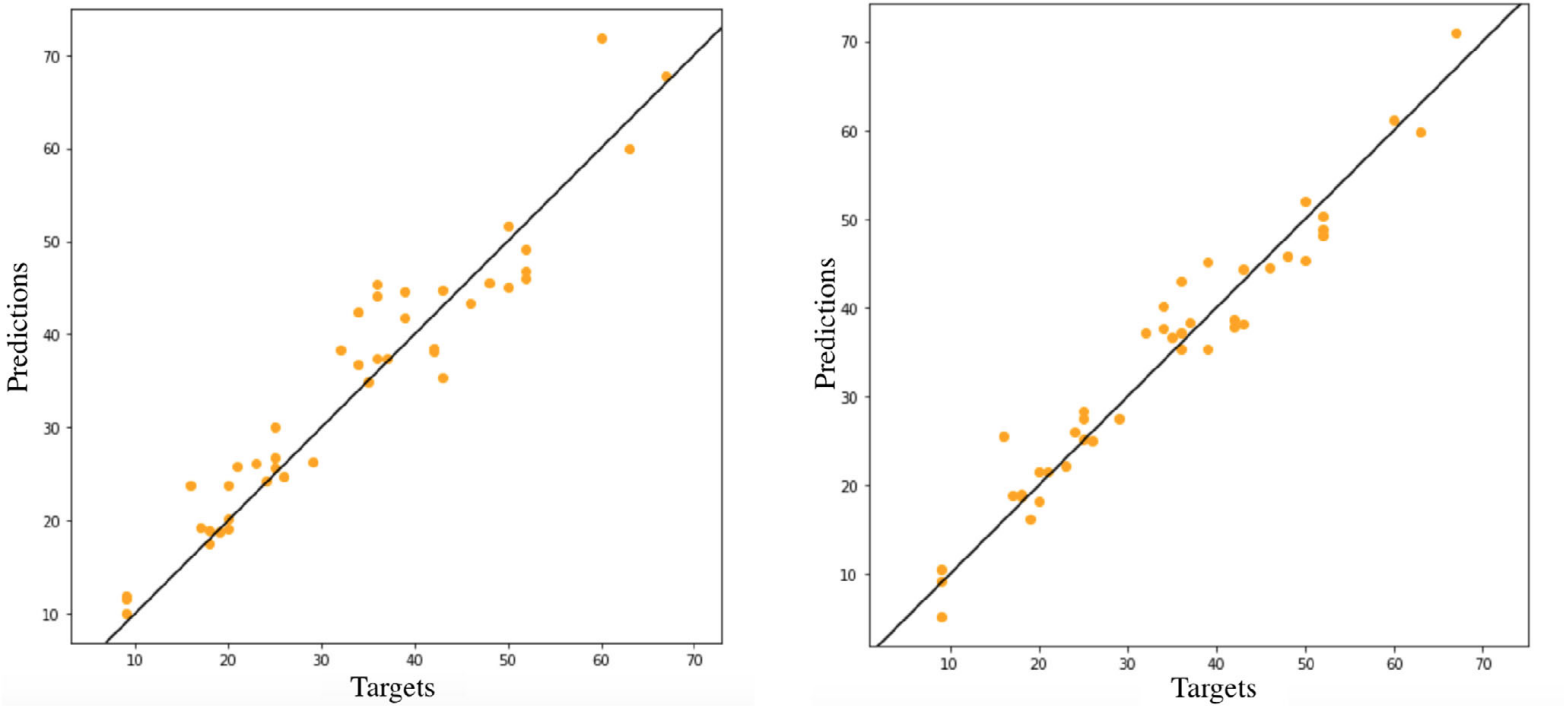
ANN model fitted with Smote and Smote+Enn on train dataset

The values of *r* ^2^ are generally lower than the ones evaluated with entire test set. This shows the difficulty of machine learning models to predict data that are less frequent during training. Ridge and elastic net show better prediction if they are fitted with imbalance dataset, while random forest is not able to predict samples in this target range. Once again, ANN is the best technique in terms of error and *r* ^2^. It is therefore evident the positive effect of balancing the dataset: SMOTE and SMOTE with ENN allow a significant improvement in model accuracy. Figure 5 shows the linear regression fitting of the model implemented by ANN technique and trained with balanced datasets. The plot shows high accuracy of the two models in the prediction of hematocrit with human standard range samples. The network fitted with both SMOTE and SMOTE with ENN training dataset are the best solutions for the prediction of samples belonging to human range of hematocrit.

**FIGURE 6:**
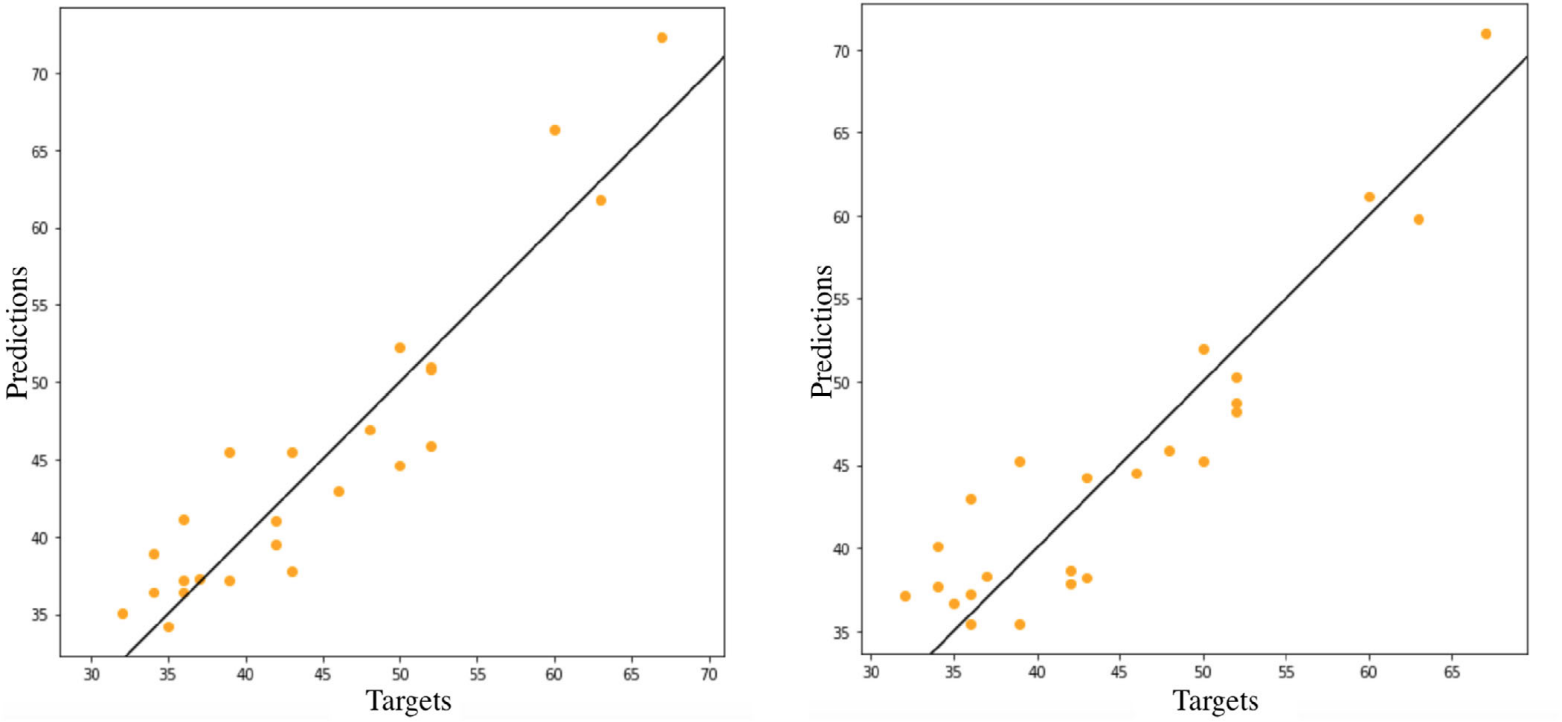
ANN model fitted with Smote and Smote+Enn on human standard range test set

## 8 CONCLUSION

Imbalanced dataset has a negative impact on the performance of machine learning. The non‐uniform distribution of samples makes harder to fit a model that works accurately for less frequent data samples. There are several methods for balancing the distribution of samples in datasets. In this work, two oversampling methods are analysed and applied on a real‐case imbalanced dataset collected for the prediction of hematocrit from animal blood spectra. SMOTE and SMOTE+ENN are applied on same dataset, resulting in two different datasets. These datasets are fitted, along with imbalance one, with different machine learning techniques to compare the performance. Both balancing techniques (SMOTE and SMOTE+ENN) do not show any advantages in terms of performances when applied on Ridge regression, elastic net or random forest. The results show an increase in performance for ANN models fitted with balanced dataset for human values of hematocrit. SMOTE or SMOTE with ENN allows the implementation of more accurate neural network models, improving the performance of machine learning models and reducing the error for the prediction of hematocrit.

Although the low number of samples, these balancing techniques allow to train ANN with higher accuracy. This is a significant advantage, because the process of acquisition new spectrum samples from bovine blood through hemodialysis test sessions is long and expensive. These results open to further investigations on the use of more sophisticated balancing algorithms, such as Adasyn that are not exploited in this work, but will be considered for further studies. Most of these advanced techniques are based on SMOTE algorithm, so we expect comparable results with the ones here reported. Collecting more data of blood samples could increase the overall results and these new datasets will require a new investigation using a similar approach in order to validate the methodology.
